# The effects of NDM-5 on *Escherichia coli* and the screening of interacting proteins

**DOI:** 10.3389/fmicb.2024.1328572

**Published:** 2024-01-30

**Authors:** Lin Li, Yiming Gao, Longbo Wang, Fang Lu, Qianyu Ji, Yanfang Zhang, Shuo Yang, Ping Cheng, Feifei Sun, Shaoqi Qu

**Affiliations:** ^1^Pharmacology and Toxicology Laboratory, Animal-Derived Food Safety Innovation Team, College of Animal Science and Technology, Anhui Agricultural University, Hefei, China; ^2^Anhui Province Key Laboratory of Veterinary Pathobiology and Disease Control, Anhui Agricultural University, Hefei, China

**Keywords:** *Escherichia coli*, NDM-5, transcriptomics, GST pull-down, mass spectrometry

## Abstract

Carbapenem-resistant *Escherichia coli* (*E. coli*) strains are widely distributed and spreading rapidly, creating significant challenges for clinical therapeutics. NDM-5, a novel mutant of New Delhi Metallo-β-Lactamase-1 (NDM-1), exhibits high hydrolase activity toward carbapenems. Since the genetic backgrounds of clinically isolated carbapenem-resistant *E. coli* are heterogeneous, it is difficult to accurately evaluate the impact of *bla*_NDM–5_ on antibiotic resistance. Herein, *E. coli* BL21 was transformed with a plasmid harboring *bla*_NDM–5_, and the resultant strain was named BL21 (pET-28a-*bla*_NDM–5_). Consistent with the findings of previous studies, the introduction of exogenous *bla*_NDM–5_ resulted in markedly greater resistance of *E. coli* to multiple β-lactam antibiotics. Compared with BL21 (pET-28a), BL21 (pET-28a-*bla*_NDM–5_) exhibited reduced motility but a significant increase in biofilm formation capacity. Furthermore, transcriptome sequencing was conducted to compare the transcriptional differences between BL21 (pET-28a) and BL21 (pET-28a-*bla*_NDM–5_). A total of 461 differentially expressed genes were identified, including those related to antibiotic resistance, such as genes associated with the active efflux system (*yddA*, *mcbR* and *emrY*), pili (*csgC*, *csgF* and *fimD*), biofilm formation (*csgD*, *csgB* and *ecpR*) and antioxidant processes (*nuoG*). Finally, the pGS21a plasmid harboring *bla*_NDM–5_ was transformed into *E. coli* Rosetta2, after which the expression of the NDM-5 protein was induced using isopropyl-β-D-thiogalactoside (IPTG). Using glutathione-S-transferase (GST) pull-down assays, total proteins from *E. coli* were scanned to screen out 82 proteins that potentially interacted with NDM-5. Our findings provide new insight into the identified proteins to identify potential antibiotic targets and design novel inhibitors of carbapenem-resistant bacteria.

## 1 Introduction

As an opportunistic pathogenic bacterium, *Escherichia coli* (*E. coli*) can cause severe infections in multiple local tissues and organs, such as the gastrointestinal tract and urinary tract, under certain conditions. The rapid dissemination of drug-resistant *E. coli* in the environment has placed extreme pressure on both livestock and human health (Steffan et al., 2020). Due to the widespread abuse of carbapenems, the emergence and transmission of carbapenem-resistant *E. coli* have become increasingly frequent. For instance, 34 of 100 urinary pathogenic *E. coli* (UPEC) strains isolated from clinical urine samples in Iran were found to be resistant to carbapenems (Khonsari et al., 2021). Carbapenem hydrolases, especially class B metalloenzymes, are mainly responsible for β-lactam resistance in *Enterobacteriaceae*. The different enzyme structures of these hydrolases can be divided into three categories, namely, B1, B2 and B3, with NDM, VIM, and IMP being the most important enzyme types (Behzadi et al., 2020; Bonnin et al., 2021).

New Delhi Metallo-β-Lactamase-1 (NDM-1), which can hydrolyze most non-monocyclic β-lactam drugs (including carbapenems), was first discovered in a *Klebsiella pneumoniae* isolate from a hospitalized Swedish patient in New Delhi (India) in 2008 (Yong et al., 2009). To date, NDM-1 has been detected in *Citrobacter*, *Salmonella*, *Shigella*, *Klebsiella*, *Enterobacteriaceae*, and *Vibrio cholerae* strains reported in Asia, Europe, Africa, North America, and Oceania (Nordmann et al., 2011a,b; Walsh et al., 2011; Berrazeg et al., 2014). As a newly emerged carbapenemase, NDM-5 has an amino acid sequence analogous to that of NDM-1, containing only two mutated sites (Val88Leu and Met154Leu). Compared with NDM-1, NDM-5 exhibits greater hydrolase activity against carbapenem, cefotaxime, cefotaxime, and ceftazidime (Rogers et al., 2013). Due to its broad hydrolysis spectrum, NDM-5 can endow host bacteria with multidrug resistance, thus causing widespread social concern.

Transcriptomics is a widely used high-throughput sequencing and analysis method that can be applied to extract comprehensive information about all transcripts in cells or tissues (Jovic et al., 2022). RNA-seq can help identify the critical factors involved in the emergence and dissemination of antibiotic resistance, facilitating the exploration of corresponding mechanisms and the screening of novel drug targets. For instance, RNA sequencing could be utilized to simultaneously evaluate the expression levels of thousands of genes under various circumstances, illustrating the mechanisms underlying the effects of multiple antibiotic resistance genes in bacteria and establishing a theoretical basis for designing novel antibiotics (Wang et al., 2009). Furthermore, Sinel et al. (2017) conducted RNA-seq analysis of *Enterococcus* plants treated with a subinhibitory concentration of daptomycin and detected alterations in the expression of 260 sRNA-encoding genes, 51% of which were correlated with carbohydrate metabolism and transport.

Interactions between bioactive proteins provide the foundation for cellular activity. Glutathione-S-transferase (GST) pull-down, which is a visual and fast method for detecting direct interactions between proteins, can be applied for the validation of interactions between known proteins and screening of unknown interacting candidates (Kim and Hakoshima, 2019). Mass spectrometry can be used to identify proteins via GST pull-down assays, playing a crucial role in the development of proteomics research. By combining GST pull-down with mass spectrometry, Lin et al. (2019) identified five proteins that interact with the porcine circovirus type 2 (PCV2) ORF4. Proteins interacting with *Helicobacter pylori* actin homologs (MreB) and influenza virus PB1-F2 were also successfully identified through a similar method (Zamarin et al., 2005; Zepeda Gurrola et al., 2017).

**Scheme 1:**
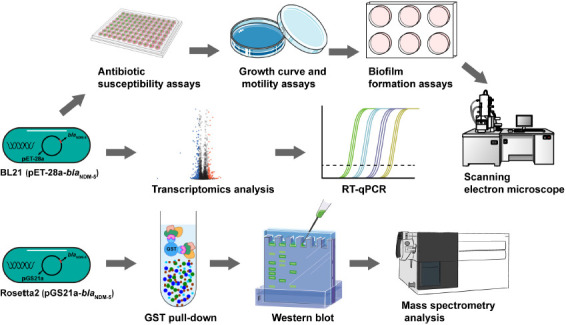
Flowchart of the experiment. The biological characteristics of BL21 (PET-28a-*bla*_NDM–5_) were determined. Transcriptomic analysis and RT-qPCR were performed to identify differentially expressed genes in BL21 (PET-28a-*bla*_NDM–5_). The expression of the NDM-5 protein was induced, after which the NDM-5-interacting proteins were identified through a GST pull-down assay and mass spectrometry.

Carbapenem-resistant bacteria isolated from clinical environments are generally resistant to both carbapenems and other antibiotics. Therefore, the *bla*_NDM–5_ resistance gene was transformed into wild-type *E. coli* to eliminate interference by environmental factors. Transcriptome sequencing was performed to analyze alterations in the gene expression levels and functional pathways related to carbapenem resistance caused by *bla*_NDM–5_. GST pull-down assays and mass spectrometry were used to identify proteins that interact with NDM-5 expressed *in vitro*, thus identifying potential drug targets for the treatment of carbapenem-resistant *E. coli* (Scheme 1).

## 2 Materials and methods

### 2.1 Wet lab section

*Bacteria and plasmids.* The plasmid pET-28a, *E. coli* ATCC25922 and the *bla*_NDM–5–_positive *E. coli* strain 120 are stored in our laboratory. *E. coli* DH5α, BL21 (DE3) competent cells (hereafter referred to as BL21), *E. coli* Rosetta2 competent cells and the pGS21a plasmid were purchased from Sangon Biotech Co., Ltd., (Shanghai, China).

*Construction of E. coli BL21 (pET-28a-bla_NDM–5_).* The *bla*_NDM–5_ DNA fragment was amplified from *E. coli* 120 by PCR. The primers used were designed with Primer Premier 5.0 according to the sequence of *E. coli bla*_NDM–5_ from GenBank (Gene ID: 2827929) (Supplementary Table 1; Zhao et al., 2023). After the amplified fragments and plasmid pET-28a were digested by *Hin*dIII/*Eco*RI and extracted by gel electrophoresis, the *bla*_NDM–5_ fragment and pET-28a were linked by Ligation Mix ligase. The recombinant plasmid was subsequently transformed into *E. coli* DH5α, after which the bacteria were cultivated on medium containing kanamycin (50 μg/mL) to construct positive recombinant plasmids. The selected plasmids were further validated by PCR and sequencing. Subsequently, the successfully constructed plasmid was transformed into BL21 competent cells by electroporation. Positive clones were identified on medium containing kanamycin (50 μg/mL) followed by PCR validation. The verified strain was named BL21 (pET-28a-*bla*_NDM–5_). Moreover, the plasmid pET-28a was electroporated into BL21 to obtain the control strain BL21 (pET-28a).

*Antibiotic susceptibility assays.* Single colonies of BL21, BL21 (pET-28a) and BL21 (pET-28a-*bla*_NDM–5_) were inoculated into 5 mL of cation-adjusted Mueller–Hinton broth (CAMHB) until the logarithmic growth period. The bacterial solution was adjusted to the 0.5 McFarland standard and diluted 100-fold. The minimum inhibitory concentrations (MICs) were determined by the micro broth dilution method according to the American Clinical and Laboratory Standards (”Clinical Lab Standards Institute. Performance standards for antimicrobial susceptibility testing.” 2016). The antibiotics were diluted twofold by CAMHB and mixed with an equal volume of bacterial suspension containing approximately 1.5 × 10^6^ colony-forming units (CFUs) mL^–1^ in a clear ultraviolet (UV)-sterilized 96-well plate. After incubation at 37°C for 18 h, the lowest concentrations of antibiotics with no visible growth of bacteria were determined as the MICs.

*Growth curve and motility assays.* One milliliter of bacterial liquid (optical density (OD)_600_ = 0.2) was diluted 100-fold and inoculated in Luria-Bertani (LB) broth medium. The absorbance of each strain at a wavelength of 600 nm was monitored by a UV spectrophotometer every 2 h to construct a growth curve.

The motility of each strain was evaluated through the following method. Bacteria were cultivated in LB broth until the OD_600_ reached 1.0. Then, 1 μL of the bacterial suspension was inoculated on the center of an LB semisolid culture plate (0.3% agar) and cultured at 37°C for 16 h (Li et al., 2021). The diameter of the growth zone was used as an index to evaluate the movement ability of the bacteria. All the experiments were repeated three times.

*Biofilm formation assays.* 200 μL of bacterial suspension (OD_600_ = 0.2) was added to one well of a UV-sterilized 96-well plate. Each strain was plated in triplicate, and an equal amount of LB broth was added to the remaining wells as the control group. Then, the 96-well plates were placed in a wet box and incubated statically at 37°C for 48 h. After the bacterial solutions had been discarded, each well was mixed with 200 μL of methanol for 15 min, followed by 3 washes with phosphate-buffered saline (PBS). Subsequently, each well was stained with 0.1% crystal violet, and then acetic acid was added to dissolve the precipitate. The OD_570_ was measured by a multifunctional microplate reader (Tecan, Switzerland). All the experiments were repeated three times.

*Scanning electron microscopy (SEM) analysis.* The bacterial solution was adjusted to the 0.5 McFarland standard and diluted 100-fold. Then, each strain was cultured in LB broth until the logarithmic growth phase. The samples were washed with PBS and fixed in glutaraldehyde overnight at 4°C. Finally, SEM was performed to observe the morphological changes in the bacteria.

*Real-time fluorescent quantitative PCR (RT-qPCR).* Seven differentially expressed genes (DEGs) related to antibiotic resistance were randomly selected from the transcriptome sequencing results, and the gene expression levels were detected via RT-qPCR. Briefly, total RNA was extracted with TRIzol reagent (Thermo Fisher Science, Carlsbad, Canada) and reverse transcribed to cDNA with a TransGen Reverse Transcription Kit (TransGen Biotech, Beijing). All the specific primers (Supplementary Table 2) used were designed according to the genome sequence of *E. coli* BL21 (NCBI reference sequence: CP020368.1). RT-qPCR was performed with the SYBR Green I method to analyze mRNA expression levels according to the instructions for the full gold quantitative PCR kit. 16S rRNA was used as a housekeeping gene to standardize the expression levels, and the relative mRNA expression data were analyzed by the 2-^△△^Ct method (Li et al., 2023). Three replicates were performed for the RT-qPCR assay.

*Expression of the NDM-5 protein and Western blot assays.* The pGS21a plasmid was digested by *Kpn*I/*EcoR* III and subsequently linked with the *bla*_NDM–5_ fragment by Ligation Mix ligase. The recombinant plasmid was transfected into *E. coli* Rosetta2. Positive colonies selected on LB medium containing 50 μg/mL ampicillin were identified by PCR and sequencing. *E. coli* Rosetta2 (pGS21a-*bla*_NDM–5_) was cultured until the OD_600_ reached 0.6. After treatment with 0.5 mmol/L isopropyl-β-D-thiogalactoside (IPTG) for 4 h, 20 μL of each bacterial culture was centrifuged, collected, mixed with 5 μL of 5 × loading buffer, and then boiled at 100°C for 10 min. The induced protein was purified following the instructions of the GST-Labeled Protein Purification Kit (Beyotime Biotechnology, Shanghai). Subsequently, the purified samples were separated via sodium dodecyl sulfate-polyacrylamide gel electrophoresis (SDS-PAGE) and transferred onto a polyvinylidene difluoride (PVDF) membrane. Then, the membrane was blocked with 5% skim milk for 2 h at room temperature and further incubated with mouse GST monoclonal primary antibodies against GST-NDM-5 and horseradish peroxidase (HRP)-conjugated goat anti-mouse secondary antibodies. Finally, the membrane was visualized and recorded by a chemiluminescence imager.

*GST pull-down assays.* Approximately 25 μg of GST and GST-NDM-5 fusion proteins were immobilized in 50 μl of 50% glutathione resin suspension and equilibrated. Total protein from *E. coli* (25 μg) was added to the immobilized GST and GST-NDM-5. After being incubated at 4°C for 4 h with gentle shaking, the mixture was centrifuged at 2000 rpm for 5 min. The precipitate was washed with PBS 3 times, after which the fusion proteins were eluted from the resin and analyzed via SDS-PAGE.

### 2.2 Dry lab section

*Transcriptomic analysis.* Single bacterial colonies were picked and cultured in LB broth overnight. A 1 mL bacterial suspension was inoculated into fresh LB broth at a ratio of 1:100 and cultured until the OD_600_ reached 0.6. Then, the cell pellets were collected by centrifugation, and total RNA was extracted with TRIzol reagent (Thermo Fisher Scientific, Carlsbad, Canada). Subsequently, the integrity, purity and concentration of the extracted RNA were evaluated by an Agilent 2100 Bioanalyzer (Agilent Technologies, Inc., California, USA). The transcriptome library was constructed according to the instructions of the Illumina TruSeq RNA Sample Prep Kit (Thermo Fisher Science, Shanghai). After the library had been qualified and optimized to achieve standards, paired-end sequencing was performed on the Illumina NovaSeq 6000 platform. The raw paired-end reads were trimmed and quality controlled by Trimmomatic. Then, the clean reads were separately aligned to the reference genome in orientation mode using Rockhopper^1^ software. If the proportion of clean reads was greater than 70%, EdgeR software was used to analyze the difference in the expression level of each transcript between samples. The DEGs were analyzed according to the fragments per kilobase of exon per million fragments mapped (FPKM) value (Behzadi and Ranjbar, 2019). Goatools^2^ was used for Gene Ontology (GO) enrichment analysis, while KOBAS^3^ was utilized for Kyoto Encyclopedia of Genes and Genomes (KEGG) pathway enrichment analysis.

*Mass spectrometry analysis.* The proteins obtained from the GST pull-down assays were digested into small peptides by pancreatin. The purified peptides were dissolved in lysis solution (0.1% formic acid and 2% acetonitrile), followed by centrifugation at 13,200 rpm and 4°C for 20 min. The supernatant was subsequently extracted for mass spectrometry analysis. The raw mass spectrometry data were retrieved from the UniProt database^4^ by MASCOT.^5^

*Statistical analysis.* All the experimental data in this study were analyzed with GraphPad Prism 8.0.2 software. The statistical significance was analyzed by the unpaired Student’s *t*-test and one-way ANOVA (*: *p* < 0.05; **: *p* < 0.01; ***: *p* < 0.001); differences with *p* < 0.05 were considered significant, those with *p* < 0.01 were considered very significant, and those with *p* < 0.001 were considered extremely significant.

## 3 Results

### 3.1 Successful construction of *E. coli* BL21 (pET-28a-*bla*_NDM–5_)

**FIGURE 1:**
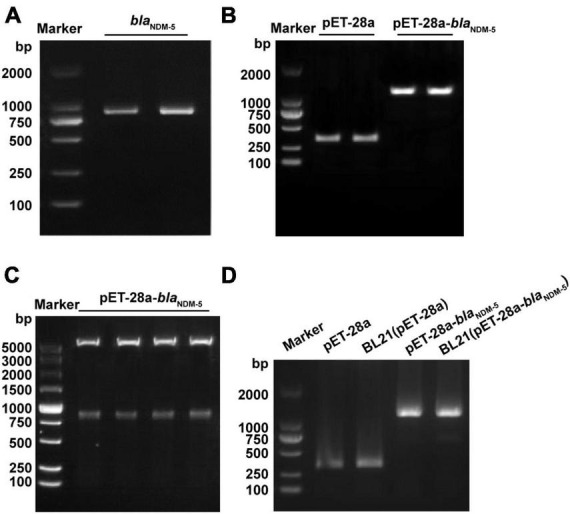
Identification of the constructed strains. **(A)** PCR amplification results of the NDM-5 gene. **(B)** PCR amplification results of the recombinant plasmid. **(C)** Construction and detection results of the pET-28a-*bla*_NDM–5_ recombinant plasmid. **(D)** PCR verification of the BL21 (pET-28a-*bla*_NDM–5_) strain.

Due to the complex genetic background of clinically isolated *bla*_NDM–5_-positive *E. coli*, the plasmid pET-28a containing the *bla*_NDM–5_ fragment was transformed into wild-type *E. coli* BL21 to exclude potential interference. After confirming the existence of *bla*_NDM–5_ in *E. coli* strain 120 by PCR (Figure 1A) and sequencing (Supplementary Table 3), the *bla*_NDM–5_ fragment was amplified and linked with plasmid pET-28a by double enzyme digestion. As shown in Figures 1B, C, the recombinant plasmid pET-28a-*bla*_NDM–5_ was successfully constructed. The sequencing data for pET-28a-*bla*_NDM–5_ are available in Supplementary Table 4. Then, *E. coli* BL21 was transfected with pET-28a-*bla*_NDM–5_, and positive colonies were screened out. Finally, PCR-amplified fragments of *E. coli* BL21 (pET-28a-*bla*_NDM–5_) were verified by electrophoresis (Figure 1D), confirming the successful construction of *E. coli* BL21 (pET-28a-*bla*_NDM–5_).

### 3.2 Increased resistance to β-lactam antibiotics in BL21 (pET-28a-*bla*_NDM–5_)

**FIGURE 2:**
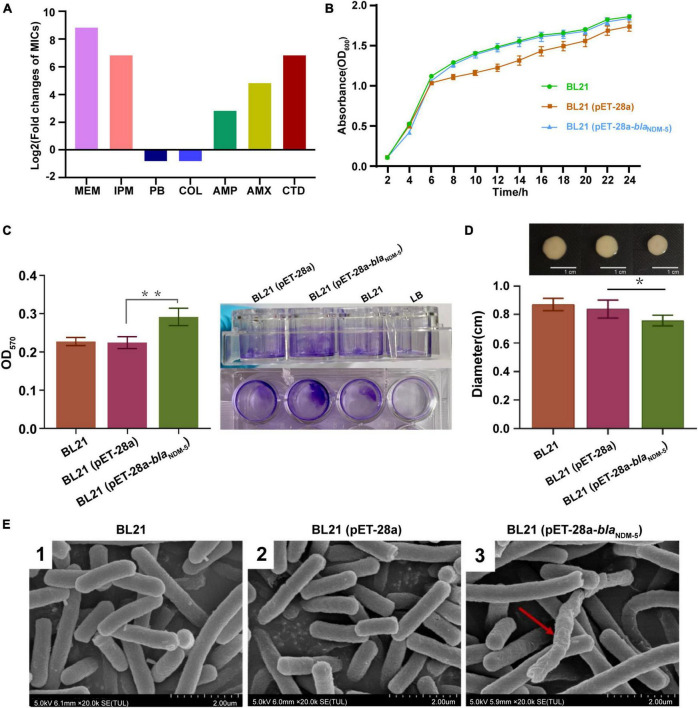
Evaluation of biological characteristics. **(A)** Antimicrobial susceptibility of the bacterial strains. **(B)** Growth curves of the strains. The growth rate was evaluated based on the OD_600_ value of the bacteria after 2, 4, 6, 8, 10, 12, 14, 16, 18, 20, 22 and 24h of incubation. **(C)** The biofilm formation ability of the strains. The OD_570_ value of the bacteria in 96-well cell culture plates measured by an enzymometer was used as an index to evaluate the biofilm formation ability of the bacteria. **p < 0.01. **(D)** Mobility of the strains. The bacterial colony diameter after 16h of incubation was used as an index to evaluate the motility of the strain. *p < 0.05. **(E)** Observation of bacterial morphology by scanning electron microscopy. Red arrows: abnormal changes.

The MICs of meropenem and imipenem against all the bacterial strains are available in Table 1. Transformation with the vector pET-28a did not change the susceptibility of BL21 to meropenem or imipenem. In contrast, the MIC of meropenem increased 520-fold (MIC = 16 μg/mL) and that of imipenem increased 128-fold (MIC = 32 μg/mL) for *bla*_NDM–5_-positive BL21. Subsequently, the susceptibility of BL21 (pET-28a) and BL21 (pET-28a-*bla*_NDM–5_) to 14 commonly used antibiotics was evaluated (Table 2). Compared with that for BL21 (pET-28a), for BL21 (pET-28a-*bla*_NDM–5_), the MIC of ceftazidime increased 128-fold and that of amoxicillin increased 32-fold, but those of colistin and polymyxin B decreased 2-fold. In addition, the susceptibility to non-β-lactam drugs such as ofloxacin and gentamicin did not significantly change after the introduction of the *bla*_NDM–5_ resistance gene. The fold changes in MICs are shown in Figure 2A.

**TABLE 1:** Results of the sensitivity of the test strains to carbapenem drugs.

Strains	MICs (μ g/mL)	
	Meropenem MEM	Imipenem IPM
BL21	0.03	0.25
BL21 (pET-28a)	0.03	0.25
BL21 (pET-28a-*bla*_*NDM*–5_)	16	32

**TABLE 2:** Antibacterial sensitivity of BL21 (pET-28a-*bla*_*NDM*–5_).

Antibacterial drugs	MICs (μ g/mL)		
	BL21	BL21 (pET-28a)	BL21 (pET-28a-*bla*_*NDM*–5_)
Tobramycin TOB	0.5	0.5	1
Polymyxin B PB	1	1	0.5
Colistin COL	1	1	0.5
Ampicillin AMP	1	1	8
Amoxicillin AMX	2	2	64
Ceftazidime CTD	0.125	0.125	16
Spectinomycin SPE	32	32	32
Gentamicin CEN	2	2	2
Amikacin AMK	2	2	2
Ciprofloxacin CIP	0.25	0.25	0.25
Cefepime FEP	<0.25	<0.25	<0.25
Levofloxacin LEV	<0.25	<0.25	<0.25
Aztreonam ATM	<0.25	<0.25	<0.25
Cefotaxime CTX	<0.25	<0.25	<0.25
Ofloxacin OFX	<0.25	<0.25	<0.25
Ennosault ENB	<0.25	<0.25	<0.25
Sulfafurazole SF	>128	>128	>128
Acetylmethaquine MEQ	<0.25	<0.25	<0.25
Florfenicol FFC	1	1	1
Tetracycline TET	2	1	1
Tigecycline TIG	<0.25	<0.25	<0.25

As shown in Figure 2B, there was no significant difference in growth rate among the three bacterial strains (*p* > 0.05), demonstrating that transformation with the *bla*_NDM–5_ recombinant plasmid did not affect the growth rate of *E. coli*. The biofilm formation ability of the strains was subsequently evaluated via the crystal violet staining method. Compared with that of BL21 and BL21 (pET-28a), the biofilm formation capacity of BL21 (pET-28a-*bla*_NDM–5_) increased markedly (*p* < 0.01) (Figure 2C). In contrast, a significant reduction in the growth zone diameter was observed for BL21 (pET-28a-*bla*_NDM–5_), indicating that the acquisition of carbapenem resistance diminishes the motility of *E. coli* BL21 (*p* < 0.05) (Figure 2D). As shown in Figure 2E, *bla*_NDM–5_ had little influence on the morphology of the bacteria. SEM revealed that only an extremely tiny fraction of the BL21 cells (pET-28a-*bla*_NDM–5_) exhibited slight deformation of cell morphology.

### 3.3 Results of transcriptome analysis

**FIGURE 3:**
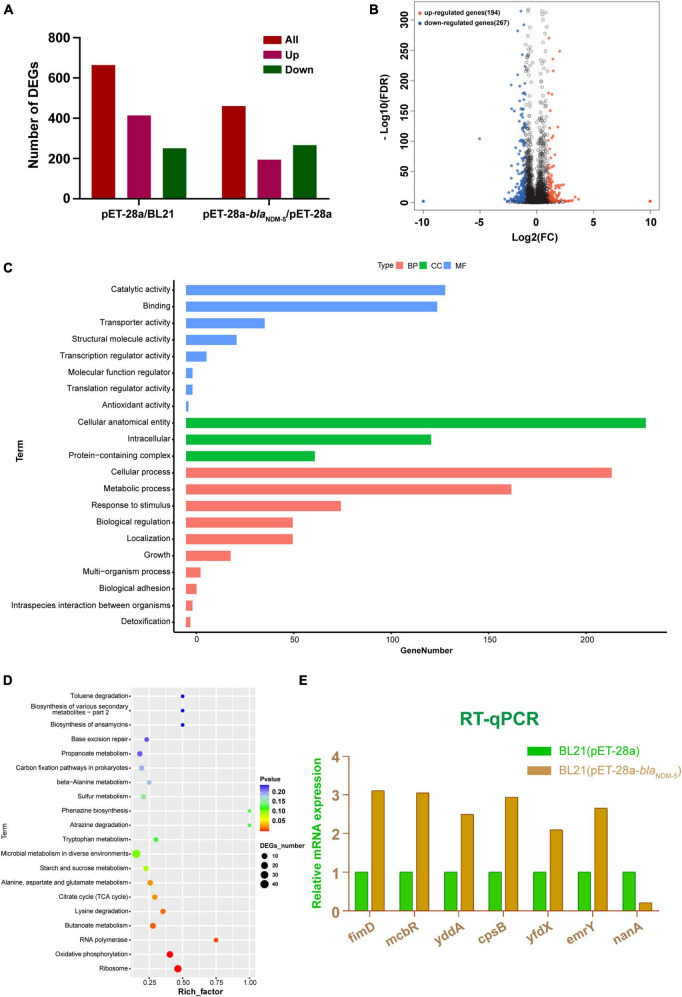
Results of transcriptome analysis. **(A)** Numbers of differentially expressed genes between groups. **(B)** Volcano map of differentially expressed genes in BL21 (pET-28a)/BL21 (pET-28a-*bla*_NDM–5_) cells. **(C)** GO classification of differentially expressed genes. **(D)** KEGG functional enrichment of the differentially expressed genes. The number of DEGs is proportional to the size of the dots. **(E)** RT-qPCR results.

DEGs were screened out as genes with | log2 (fold change)| > 1 and *Q*-value < 0.005. A total of 665 DEGs were detected in the BL21 (pET-28a)/BL21 group, including 414 upregulated genes and 251 downregulated genes. Moreover, 461 DEGs, including 194 upregulated genes and 267 downregulated genes, were detected in the BL21 (pET-28a)/BL21 (pET-28a-*bla*_NDM–5_) group (Figure 3A). The distribution of DEGs in the BL21 (pET-28a)/BL21 (pET-28a-*bla*_NDM–5_) group is shown in Figure 3B. The genes related to antibiotic resistance were selected from the identified DEGs, and some of them are listed in Supplementary Table 5. These genes included active efflux system genes (*yddA*, *mcbR* and *emrY*), pilus genes (*csgC*, *csgF* and *fimD*), biofilm formation genes (*csgD*, *csgB* and *ecpR*) and antioxidant process-related genes (*nuoG*).

GO enrichment classification was used to annotate the functional associations of the genes. A total of 307 DEGs from the BL21 (pET-28a-*bla*_NDM–5_)/BL21 (pET-28a) group were annotated into three categories: biological process (BP), cellular component (CC), and molecular function (MF). As shown in Figure 3C, DEGs in the biological process category were mainly enriched in the cellular process, metabolic process, response to stimulus, biological regulation, and cellular localization terms. In the cellular component category, DEGs were enriched in terms such as intracellular, protein-containing complexes and cellular antioxidant entities. In addition, in the molecular function category, terms related to catalytic activity, transport activity, structural molecule activity and binding reactions were foremost highly enriched by the DEGs. Subsequently, the KEGG database was used to annotate the DEGs into 99 pathways, and they were chiefly enriched in ribosome, oxidative phosphorylation, RNA polymerase, butyrate metabolism, lysine degradation, the tricarboxylic acid cycle, alanine metabolism and other functional pathways (Figure 3D).

To explore the molecular mechanism underlying the effect of *bla*_NDM–5_, 7 genes were randomly selected from among the DEGs associated with antibiotic resistance, and these genes were subjected to RT-qPCR verification. Compared with those in BL21 (pET-28a), in BL21 (pET-28a-*bla*_NDM–5_), the expression levels of *fimD*, *mcbR*, *cpsB*, *yddA*, *yfdX*, and *emrY* were significantly upregulated, while the expression level of *nanA* was significantly downregulated (Figure 3E). These results are consistent with the outcomes of transcriptome sequencing, confirming the reliability of the transcriptome sequencing data.

### 3.4 82 NDM-5-interacting proteins were screened out and analyzed via mass spectrometry

**FIGURE 4:**
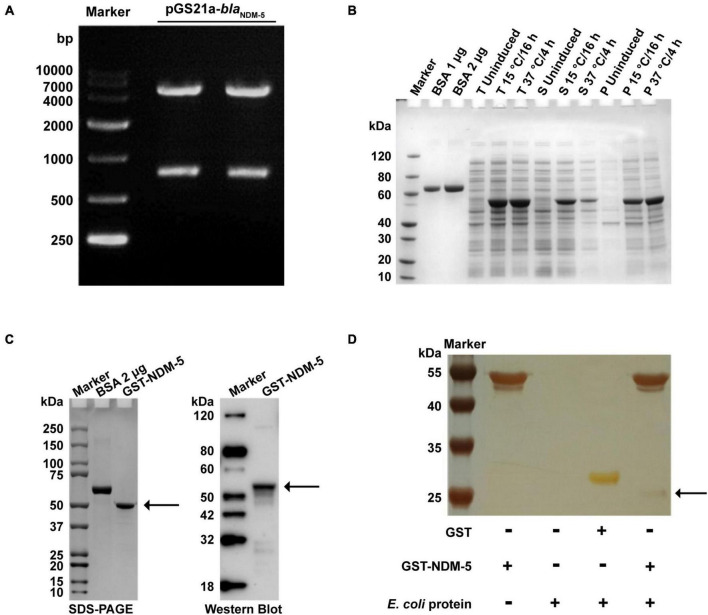
Expression and validation of the NDM-5 protein and screening of its interacting proteins. **(A)** Verification of the recombinant plasmid pGS21a-NDM-5. **(B)** Detection of recombinant protein expression. BSA: bovine serum albumin; T: total bacteria; S: supernatant; P: precipitate. **(C)** Identification of the recombinant protein after purification. The black arrows indicate the NDM-5 protein. **(D)** Silver stain analysis. The black arrow indicates interacting proteins in the GST-NDM-5 + /*E. coli* group.

Double enzyme-digested fragments of pGS21a-*bla*_NDM–5_ were verified by gel electrophoresis (Figure 4A). The sequencing results are available in Supplementary Table 6. After pGS21a-*bla*_NDM–5_ was transformed into *E. coli* Rosetta2, IPTG was used to induce protein expression. As expected, SDS-PAGE analysis detected a specific band at 54 kDa, indicating successful expression of the NDM-5 protein (Figure 4B). Subsequently, the NDM-5 protein was purified and linked with GST labels. As shown in Figure 4C, a single and distinct band could be observed at 54.5 kDa by SDS-PAGE, which is consistent with the results of Western blot analysis.

GST pull-down assays were performed for GST-NDM-5 and total *E. coli* proteins to investigate the proteins potentially interacting with NDM-5. The purified proteins were verified by SDS-PAGE. In addition to the NDM-5 band at 54.5 kDa, other protein bands were also observed, demonstrating the presence of NDM-5-interacting proteins (Figure 4D). The interacting proteins were extracted and purified, followed by mass spectrometry identification. A total of 665 interacting proteins were identified in the GST + /*E. coli* + control group, and 466 potential interacting proteins were identified in the GST-NDM-5 + /*E. coli* + interacting group. Since 384 proteins were detected in both groups, these proteins were removed from the GST-NDM-5 + /*E. coli* + group, leaving the remaining 82 proteins as candidates that potentially interact with NDM-5 (Supplementary Figure 1). Some of the identified NDM-5-interacting proteins are listed in Supplementary Table 7.

**FIGURE 5:**
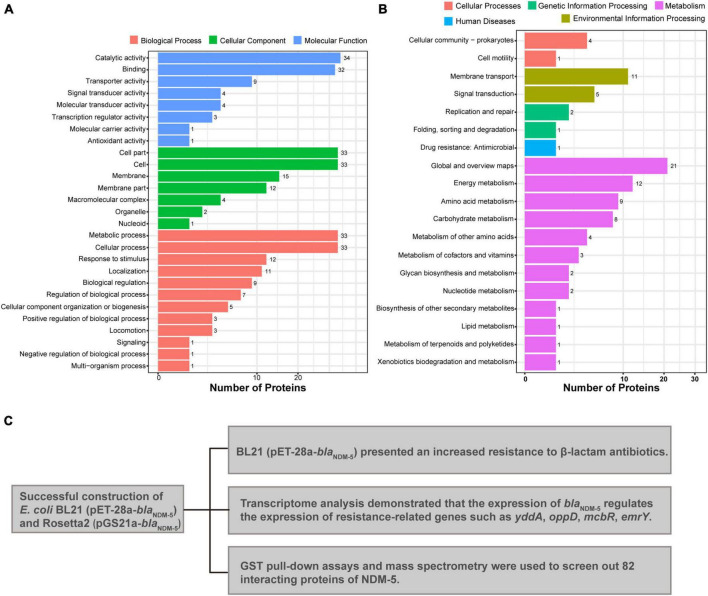
Mass spectrometry analysis results. **(A)** GO annotation of potential NDM-5-interacting proteins. **(B)** KEGG annotation of potential NDM-5-interacting proteins. **(C)** Summary of the effects of NDM-5 on *E. coli* and the screening of interacting proteins.

GO and KEGG analyzes were performed to elucidate the biological function of the 82 interacting proteins. A total of 307 GO-enriched functional pathways were annotated. Among them, 88 pathways were involved in molecular functions, mainly binding, catalytic and transport activities. In addition, 100 pathways were correlated with cellular components, including cells, cellular components and macromolecular complexes. The remaining 119 pathways were related to biological processes, such as metabolic processes, cellular processes, localization processes, biological regulation processes and responses to stimuli (Figure 5A). Moreover, 90 enriched functional pathways were annotated by KEGG analysis, including 65 metabolic pathways, 16 environmental information processing pathways, and 3 genetic information processing pathways (Figure 5B).

## 4 Discussion

Carbapenem resistant *E. coli* strains usually contain other resistance genes. Therefore, herein, we used the pET-28a plasmid to transform *bla*_NDM–5_ into wild-type *E. coli* to exclude interference by other resistance genes. Compared with the wild type, the BL21 (pET-28a-*bla*_NDM–5_) strain exhibited markedly increased resistance to meropenem (520-fold) and imipenem (128-fold) coupled with markedly increased resistance to other β-lactam antibiotics, such as amoxicillin and ceftazidime. Meanwhile, the MICs of non-β-lactam antibiotics did not change, indicating that NDM-5 cannot hydrolyze non-β-lactam drugs. However, twofold decreases in the MICs of colistin and polymyxin B were observed after the introduction of *bla*_NDM–5_. This increase in susceptibility could be attributed to the downregulation of *eptB* expression since the chromosomally encoded PEA (phosphatidyl-ethanolamine) transferases (e.g., EptA, EptB and EptC) can mediate polymyxin resistance in gram-negative bacteria through the modification of Lipid A (Huang et al., 2018; Supplementary Table 5).

Our results indicated that the transformation of pET-28a-*bla*_NDM–5_ marginally reduced the growth rate of BL21, significantly restricted bacterial motility and strongly enhanced biofilm formation capacity. It is speculated that the acquisition of carbapenem resistance is probably caused by these phenotypic changes. For instance, compared with those of susceptible strains, the motility and growth rate of polymyxin-resistant bacteria are significantly lower (Tietgen et al., 2019; Yang et al., 2020). In addition, biofilms can prevent antibiotics from penetrating cell membranes and entering the interior of cells, thus enhancing bacterial resistance to multiple antibiotics (Romeu et al., 2020). Lee et al. (2020) transformed plasmids harboring *bla*_NDM–1_ and *bla*_*OXA–*232_ into *E. coli* DH5α and reported that both transformants had a significantly enhanced biofilm formation capacity.

The transcriptome analysis results indicated that the expression of biofilm-related genes (*ycgZ*, *csgD* and *mcbR*) was significantly upregulated, which is consistent with the observed bacterial phenotypes. Therefore, it is speculated that *csgD* and *mcbR* increase bacterial resistance through the modulation of motility and biofilm formation. The CSG region encodes pilus-related genes, while CsgD controls cell aggregation and inhibits flagellar synthesis through direct regulation of Curli pili (Herrington and Sitaras, 2013; Behzadi and Behzadi, 2017; Ogasawara et al., 2020). In addition, McbR can regulate the formation of biofilms and mucus by inhibiting the expression of the periplasmic protein YbiM and binding to the *yciG* promoter to activate the transcription of the *yciGFE* operon in *E. coli* K-12 (Yu et al., 2019). We also identified several efflux pump-related genes, including *yddA*, *mcbR*, *emrY* and *mdtl*. The efflux pump can expel intracellular antibiotics to reduce the effective concentration of drugs, thus conferring antibiotic resistance to bacteria (Lee et al., 2017). As a prokaryotic homolog of P-gp, YddA is a representative member of the superfamily of ATP-binding cassette (ABC) transporters that can recognize and transport a variety of substrates (Feng et al., 2020). In avian pathogenic *E. coli*, an electrophoretic mobility shift assay (EMSA) was used to confirm that McbR could specifically bind to the promoter regions of *acrAB*, *acrD*, *acrR*, *emrD* and *mdtD*, revealing that McbR modulates bacterial resistance by directly activating the transcription of efflux pump genes (Yu et al., 2020). Furthermore, the amino acid sequence of EmrY shares 63.3% homology with that of EmrB, and these proteins together constitute a multidrug efflux pump (Tanabe et al., 1997; Wei et al., 2022). The RNA-seq results verified the upregulated expression of such efflux pump genes, partially explaining the correlation between *bla*_NDM–5_ and carbapenem resistance. pET-28a is a plasmid encoding an RNA polymerase, an *ori* gene, a *lacI* gene, and an antimicrobial gene. Accordingly, protein expression of BL21 (pET-28a) and BL21 alone should be similar. However, a total of 665 DEGs were detected in the BL21 (pET-28a)/BL21 group, which is quite different from the expected results and is one of the limitations of this study.

Due to the high solubility, distinct affinity and high-efficiency expression of GST tags, GST fusion proteins are commonly utilized for the identification of interacting proteins (Pan et al., 2011). *E. coli* grows best at 37°C, but at that temperature, the expression of specific proteins may not reach the optimal level due to “leakage” of some expression vectors and metabolic pressure on bacterial cells (Sørensen and Mortensen, 2005). Low temperature can slow the synthesis rate of proteins and alter the dynamics of peptide folding to increase the possibility of correct folding, thus facilitating the soluble expression of fusion proteins (Donovan et al., 2000; Sadraeian et al., 2013). In this paper, we compared the concentration ratios of soluble proteins at 15°C and 37°C and determined that 15°C was the optimal incubation temperature for the expression of NDM-5. Subsequently, 82 proteins that interact with NDM-5 were identified by GST pull-down assays. Among them, the transcriptional regulator AraC, the multidrug efflux RND transporter permease AcrD, and the multidrug efflux transporter outer membrane subunit MdtP are closely associated with the function of efflux pumps. In gram-negative bacteria, the overexpression of the AcrAB, AcrD and MdtABC efflux pumps can cause antibiotic resistance (Yu and Schneiders, 2012). Therefore, the upregulated expression of efflux pump-related genes detected by transcriptome sequencing is presumably caused by the interaction between the NDM-5 protein and efflux pump-related proteins. The detected histidine kinase precursor ZraS and histidine kinase YpdA are two-component system sensors that participate in various cellular processes, including antibiotic resistance, quorum sensing and osmotic pressure (Zschiedrich et al., 2016). It is speculated that NDM-5 could interact with a two-component system to regulate the expression of antibiotic resistance genes, facilitating the recognition of the host environment and adaptation to adverse environments. ABC transporters are a family of proteins with highly conserved transmembrane structures that transport substances through the energy generated by ATP hydrolysis (Thomas et al., 2020). The screened proteins related to ABC transporters, such as A0A140N5T4, A0A140NBB8 and A0A140NFJ3, may interact with NDM-5 to participate in the regulation of bacterial resistance. The major limitations of the present study are that the proteins potentially interacting with NDM-5 were screened, and the mechanism of interaction could not be accurately investigated (Figure 5C). Considerably more work is needed to identify proteins that interact with NDM-5, with the aim of providing a theoretical basis for exploring new targets for antimicrobial drugs, identifying new inhibitors against carbapenem-resistant *E. coli*, and preventing and controlling superresistant bacteria. Although the potential mechanisms through which NDM-5 interacts with proteins need further investigation, the identified interacting proteins provide an outstanding foundation for exploring the function of NDM-5 in bacteria.

## 5 Conclusion

In this study, we used the wild-type strain of *E. coli* and the plasmid pET-28a to construct a *bla*_NDM–5–_positive strain. The antibiotic susceptibility and biological characteristics of *E. coli* BL21 (pET-28a-*bla*_NDM–5_) were evaluated, revealing that *bla*_NDM–5_ has a significant impact on the bacterial phenotype. Transcriptome analysis and RT-qPCR demonstrated that *bla*_NDM–5_ could cooperate with other antibiotic resistance genes (*yddA*, *oppD*, *mcbR*, *emrY*, etc.) to increase bacterial resistance to various β-lactam antibiotics, especially imipenem and meropenem. We successfully expressed the soluble NDM-5 protein *in vitro* and screened 82 proteins that interact with NDM-5. In conclusion, our study could provide a theoretical basis for the exploration of novel drug targets, but further research focused on the interaction mechanism between specific proteins and NDM-5 is needed.

## Data availability statement

The original contributions presented in the study are publicly available. This data can be found here: Raw picture: https://www.jianguoyun.com/p/DbW-QHwQh8GRDBiflaUFIAA. Results of transcriptome analysis: https://www.jianguoyun.com/p/DeBSq18Qh8GRDBihlaUFIAA. Raw data: https://www.jianguoyun.com/p/Df8zlmAQh8GRDBiilaUFIAA.

## Author contributions

LL: Resources, Project administration, Supervision, Writing – review and editing. YG: Writing – original draft, Methodology, Investigation, Conceptualization. LW: Data curation, Formal Analysis, Writing – original draft, Writing – review and editing. FL: Methodology, Project administration, Writing – original draft. QJ: Project administration, Software, Writing – review and editing. SY: Writing – review and editing, Conceptualization. YZ: Data curation, Writing – review and editing. PC: Resources, Writing – review and editing. FS: Validation, Methodology, Writing – review and editing, Supervision. SQ: Validation, Supervision, Software, Writing – review and editing.

## Footnotes

1
https://cs.wellesley.edu/~btjaden/Rockhopper/

2
https://github.com/tanghaibao/GOatools

3
http://kobas.cbi.pku.edu.cn/home.do

4
https://www.uniprot.org/taxonomy/469008

5
http://www.matrixscience.com/
