# Supplementary material for: The effects of NDM-5 on Escherichia coli and the screening of interacting proteins
Source: Front Microbiol. 2024 Jan 30;15:1328572. doi: 10.3389/fmicb.2024.1328572 (PMC10861311; doi:10.3389/fmicb.2024.1328572)
Supplement: Supplementary file 1 [file Data_Sheet_1.doc]

Supplementary Material

**The effects of NDM-5 on *Escherichia coli* and the screening of interacting proteins**

**Lin Li^†1,2^, Yiming Gao ^†1^, Longbo Wang^1^, Fang Lu^1^, Qianyu Ji^1^, Yanfang Zhang^1^, Shuo Yang^1^, Ping Cheng^1,2^, Feifei Sun ^1,2*^, Shaoqi Qu^1,2*^**

**^1^**Pharmacology and Toxicology Laboratory, Animal-derived food safety innovation team, College of Animal Science and Technology, Anhui Agricultural University, Hefei, China

**^2^**Anhui Province Key Lab of Veterinary Pathobiology and Disease Control, Anhui Agricultural University, Hefei, China

*** Correspondence:**

Feifei Sun ^1,2^**^*^**

13141212481163@com

Shaoqi Qu^1,2*^

sqq@ahau.edu.cn

Lin Li^†1,2^ and Yiming Gao ^†1^ contributed equally to this work and share the first authorship.

**Keywords: *Escherichia coli*; NDM-5; Transcriptomics; GST pull-down; Mass spectrometry.**

**Supplemental Figure**

**
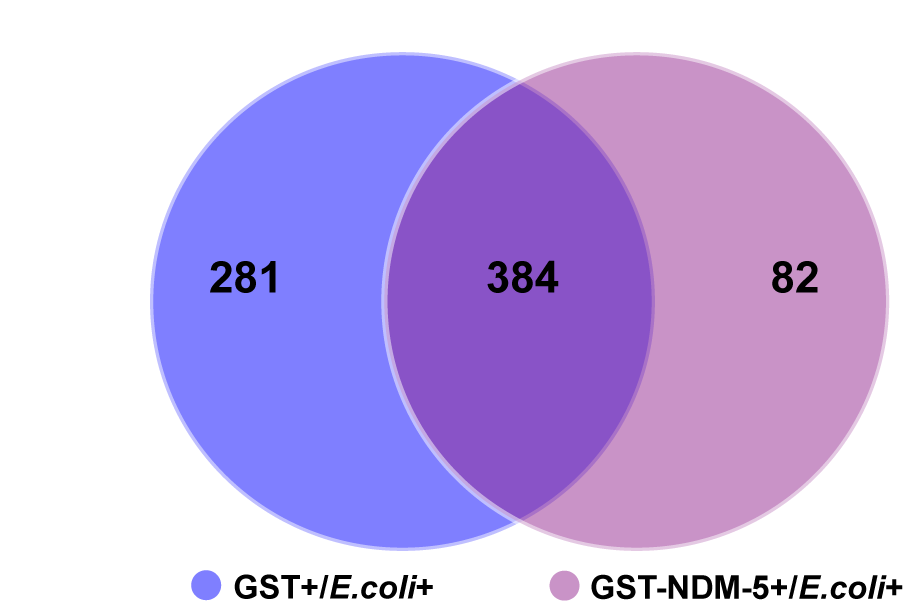
**

Figure 1. Wayne plot of differential protein between the GST pull-down experimental and control groups

**Supplemental Tables**

**Supplemental Table 1.** **Primer sequence**

| Primers | Sequence(5’→3’) | Tm/°C | Product/bp |
| --- | --- | --- | --- |
| NDM-5-F1 | TCAGCGCAGCTTGTCGGCC | 68 | 813 |
| NDM-5-R1 | ATGGAATTGCCCAATATTATGCACC |
| pET-28a-F | TAATACGACTCACTATAGGG | 57.5 | 318 |
| pET-28a-R | GCTAGTTATTGCTCAGCGG |
| NDM-5-F2 | CCCAAGCTTTCAGCGCAGCTTGTCGGCC | 68 | 825 |
| NDM-5-R2 | CCGGAATTCATGGAATTGCCCAATATTATGCACC |
| pGS21a-F | CACAAATTGATAAGTACTTG | 60 | 264 |
| pGS21a-R | GCACACCAGCTATCTCGACA |

**Supplemental Table 2. Real-time quantitative PCR primer sequence**

| Gene | Forward sequence (5’-3’) | Reverse sequence (5’-3’) |
| --- | --- | --- |
| *fimD* | GCCTCAGATGACAATATGTTACC | CAGCCTCTTTGATCGTTACCT |
| *mcbR* | CCGCTACGAACTGGAATTAATGG | CACACAGGATGGGCATATTTGAG |
| *yddA* | CAATCTGGGCGGACTGATGAA | GTTGATGGAACTCATACAAGCGAT |
| *cpsB* | CGTGTGGATGAAGAAGCGTTTC | GAAGACCAAGAACCGACATCACT |
| *yfdX* | CAACTGTATGGGCTGCTGATAA | TCACGCATCGCATATAAACCTT |
| *emrY* | TTATTAAGGAATTACCCACCAGAAA | CGCCCTTTATCAAGCATAATTTG |
| *nanA* | TCCTGATCTTGTGCTCTATAACG | CGCCAGCCCATGATGTTG |
| 16S rRNA | GTGCCAGCMGCCGCGG | GGACTACHVGGGTWTCTAAT |

**Supplemental Table 3. Sequencing results of PCR products using NDM-5-F1/NDM-5-R1 as primers (identification of *E. coli* *bla*_NDM-5_ gene)**

| TCAGCGCAGCTTGTCGGCCATGCGGGCCGTATGAGTGATTGCGGCGCGGCTATCGGGGGCGGAATGGCTCATCACGATCATGCTGGCCTTGGGGAACGCCGCACCAAACGCGCGCGCTGACGCGGCGTAGTGCTCAGTGTCGGCATCACCGAGATTGCCGAGCGACTTGGCCTTGCTGTCCTTGATCAGGCAGCCACCAAAAGCGATGTCGGTGCCGTCGATCCCAACGGTGATATTGTCACTGGTGTGGCCGGGGCCGGGGTAAAATACCTTGAGCGGGCCAAAGTTGGGCGCGGTTGCTGGTTCGACCCAGCCATTGGCGGCGAAAGTCAGGCTGTGTTGCGCCGCAACCAGCCCCTCTTGCGGGGCAAGCTGGTTCGACAACGCATTGGCATAAGTCGCAATCCCCGCCGCATGCAGCGCGTCCATACCGCCCATCTTGTCCTGATGCGCGTGAGTCACCACCGCCAGCGCGACCGGCAGGTTGATCTCCTGCTTGATCCAGTTGAGGATCTGGGCGGTCTGGTCATCGGTCCAGGCGGTATCGACCAACAGCACGCGGCCGCCATCCCTGACGATCAAACCGTTGGAAGCGACTGCCCCGAAACCCGGCATGTCGAGATAGGAAGTGTGCTGCCAGACATTCGGTGCGAGCTGGCGGAAAACCAGATCGCCAAACCGTTGGTCGCCAGTTTCCATTTGCTGGCCAATCGTCGGGCGGATTTCACCGGGCATGCACCCGCTCAGCATCAATGCAGCGGCTAATGCGGTGCTCAGCTTCGCGACCGGGTGCATAATATTGGGCAATTCCAT |
| --- |

# Supplemental Table 4. PCR product sequencing results using pET-28a-F/pET-28a-R as primers (pET-28a-*bla*_NDM-5_ recombinant plasmid)

| TAATACGACTCACTATAGGGGAATTGTGAGCGGATAACAATTCCCCTCTAGAAATAATTTTGTTTAACTTTAAGAAGGAGATATACCATGGGCAGCAGCCATCATCATCATCATCACAGCAGCGGCCTGGTGCCGCGCGGCAGCCATATGGCTAGCATGACTGGTGGACAGCAAATGGGTCGCGGATCCGAATTCTCAGCGCAGCTTGTCGGCCATGCGGGCCGTATGAGTGATTGCGGCGCGGCTATCGGGGGCGGAATGGCTCATCACGATCATGCTGGCCTTGGGGAACGCCGCACCAAACGCGCGCGCTGACGCGGCGTAGTGCTCAGTGTCGGCATCACCGAGATTGCCGAGCGACTTGGCCTTGCTGTCCTTGATCAGGCAGCCACCAAAAGCGATGTCGGTGCCGTCGATCCCAACGGTGATATTGTCACTGGTGTGGCCGGGGCCGGGGTAAAATACCTTGAGCGGGCCAAAGTTGGGCGCGGTTGCTGGTTCGACCCAGCCATTGGCGGCGAAAGTCAGGCTGTGTTGCGCCGCAACCAGCCCCTCTTGCGGGGCAAGCTGGTTCGACAACGCATTGGCATAAGTCGCAATCCCCGCCGCATGCAGCGCGTCCATACCGCCCATCTTGTCCTGATGCGCGTGAGTCACCACCGCCAGCGCGACCGGCAGGTTGATCTCCTGCTTGATCCAGTTGAGGATCTGGGCGGTCTGGTCATCGGTCCAGGCGGTATCGACCAACAGCACGCGGCCGCCATCCCTGACGATCAAACCGTTGGAAGCGACTGCCCCGAAACCCGGCATGTCGAGATAGGAAGTGTGCTGCCAGACATTCGGTGCGAGCTGGCGGAAAACCAGATCGCCAAACCGTTGGTCGCCAGTTTCCATTTGCTGGCCAATCGTCGGGCGGATTTCACCGGGCATGCACCCGCTCAGCATCAATGCAGCGGCTAATGCGGTGCTCAGCTTCGCGACCGGGTGCATAATATTGGGCAATTCCATAAGCTTGCGGCCGCACTCGAGCACCACCACCACCACCACTGAGATCCGGCTGCTAACAAAGCCCGAAAGGAAGCTGAGTTGGCTGCTGCCACCGCTGAGCAATAACTAGC |
| --- |

# Supplemental Table 5. Partial differentially expressed genes

| Gene name | Product function | log2FC |
| --- | --- | --- |
|  |  | K1/K |
| *ycgZ* | RcsB connector protein for regulation of biofilm and acid-resistance | 3.3792 |
| *csgF* | curli nucleation outer membrane protein | 2.7900 |
| *ompL* | outer membrane porin L | 2.56384 |
| *csgD* | csgBAC operon transcriptional regulator | 2.4451 |
| *csgC* | curli assembly protein | 1.6871 |
| *nuoG* | NADH:ubiquinone oxidoreductase, chain G | 1.5613 |
| *fimD* | fimbrial usher outer membrane porin protein | 1.5566 |
| *emrY* | putative multidrug efflux system | 1.3279 |
| *ecpR* | putative transcriptional regulator for the ecp operon | 1.2284 |
| *mcbR* | colanic acid and biofilm gene transcriptional regulator, MqsR-controlled | 1.213652952 |
| *yddA* | putative multidrug ABC transporter permease/ATPase | 1.086550032 |
| *cpsB* | mannose-1-phosphate guanyltransferase | 1.471583936 |
| *oppD* | ABC transporter ATP-binding protein | -1.234759085 |
| *sapC* | antimicrobial peptide transport ABC transporter permease | -1.062938169 |
| *mdtG* | multidrug resistance protein MdtG | -1.011071785 |
| *yehY* | inner membrane putative ABC superfamily transporter permease | -1.070389328 |
| *eptB* | KDO phosphoethanolamine transferase, Ca(2+)-inducible | -1.049948295 |
| *glnQ* | glutamine transport ATP-binding protein GlnQ | -1.294537823 |

K：BL21 (pET-28a)；K1：BL21 (pET-28a-*bla*_NDM-5_）；FC: Fold Change.

**Supplemental Table 6. Sequencing Results of PCR Products Using pGS21a-F/pGS21a-R as Primers (pGS21a-*bla*_NDM-5_ recombinant plasmid)**

| ATGTCTGGTTCTCATCATCATCATCATCATAGCAGCGGTATGTCCCCTATACTAGGTTATTGGAAAATTAAGGGCCTTGTGCAACCCACTCGACTTCTTTTGGAATATCTTGAAGAAAAATATGAAGAGCATTTGTATGAGCGCGATGAAGGTGATAAATGGCGAAACAAAAAGTTTGAATTGGGTTTGGAGTTTCCCAATCTTCCTTATTATATTGATGGTGATGTTAAATTAACACAGTCTATGGCCATCATACGTTATATAGCTGACAAGCACAACATGTTGGGTGGTTGTCCAAAAGAGCGTGCAGAGATTTCAATGCTTGAAGGAGCGGTTTTGGATATTAGATACGGTGTTTCGAGAATTGCATATAGTAAAGACTTTGAAACTCTCAAAGTTGATTTTCTTAGCAAGCTACCTGAAATGCTGAAAATGTTCGAAGATCGTTTATGTCATAAAACATATTTAAATGGTGATCATGTAACCCATCCTGACTTCATGTTGTATGACGCTCTTGATGTTGTTTTATACATGGACCCAATGTGCCTGGATGCGTTCCCAAAATTAGTTTGTTTTAAAAAACGTATTGAAGCTATCCCACAAATTGATAAGTACTTGAAATCCAGCAAGTATATAGCATGGCCTTTGCAGGGCTGGCAAGCCACGTTTGGTGGTGGCGACCATCCTCCAAAATCGGATCTGGGCCACACAGGCCATAGATCTGGTACCGAGAATTTGTACTTTCAGGGTGAGATTAGACCTACCATCGGTCAGCAAATGGAAACCGGCGACCAACGTTTTGGCGACCTGGTCTTTCGTCAGCTGGCGCCAAATGTTTGGCAGCACACCAGCTATCTCGACATGCCGGGTTTTGGTGCTGTAGCATCTAACGGCTTGATCGTTCGTGATGGCGGCCGCGTTCTGCTGGTAGATACCGCATGGACCGATGATCAGACCGCGCAAATTCTGAACTGGATTAAGCAAGAGATCAACTTGCCCGTGGCGCTGGCTGTGGTGACTCACGCCCATCAGGATAAGATGGGTGGTATGGATGCCTTGCATGCTGCGGGTATTGCTACGTACGCGAATGCGCTATCCAATCAGCTTGCACCGCAAGAAGGCCTCGTTGCAGCTCAACATAGCCTGACCTTCGCCGCGAACGGTTGGGTTGAACCGGCGACCGCACCGAACTTTGGTCCGCTGAAGGTGTTCTACCCGGGACCGGGCCACACGAGCGACAACATCACCGTTGGCATCGACGGCACCGATATCGCGTTCGGTGGCTGCCTGATTAAAGATAGTAAGGCCAAATCCCTGGGCAACTTGGGCGACGCGGACACCGAGCATTATGCCGCGTCTGCACGTGCCTTCGGTGCAGCATTCCCAAAGGCGTCCATGATTGTTATGAGTCATAGCGCGCCGGATAGCCGAGCGGCCATAACCCACACGGCTCGTATGGCAGACAAATTACGTTAATGAAAGCTT |
| --- |

# Supplemental Table 7. NDM-5 interacting partial proteins screened by GST pull-down

| Protein No. | Gene name | Protein annotation |
| --- | --- | --- |
| C5W865 | *cysI* | Sulfite reductase [NADPH] hemoprotein beta-component |
| A0A140NHQ0 | *metF* | Methylenetetrahydrofolate reductase |
| A0A140NGE3 | *nhaR* | Transcriptional regulator, LysR family |
| A0A140NG17 | *robA* | Transcriptional regulator, AraC family |
| A0A140NFK9 | *zraS* | histidine kinase precursor |
| A0A140NFJ7 | *yigL* | Cof-like hydrolase |
| A0A140NFJ3 | *tauB* | ABC transporter related precursor |
| A0A140NFE5 | *dinB* | DNA polymerase IV |
| A0A140NF93 | ECBD_3391 | OmpA/MotB domain protein |
| A0A140NEM3 | *frdC* | Fumarate reductase subunit C |
| A0A140NEI5 | *rhsD* | YD repeat protein |
| A0A140NEB2 | *dapB* | 4-hydroxy-tetrahydrodipicolinate reductase |
| A0A140NDW1 | *pflA* | Pyruvate formate-lyase-activating enzyme |
| A0A140NDN8 | *cra* | Catabolite repressor/activator |
| A0A140NDN0 | *ddlB* | D-alanine--D-alanine ligase |
| A0A140NDE7 | *fhuF* | Ferric iron reductase |
| A0A140ND64 | ECBD_3334 | Carbamate kinase |
| A0A140ND48 | ECBD_4277 | Transcriptional regulator, LacI family |
| A0A140ND06 | *mhpF* | Acetaldehyde dehydrogenase |
| A0A140NCM1 | *metB* | O-succinylhomoserine (Thiol)-lyase |
| A0A140NCJ7 | *argH* | Argininosuccinate lyase |
| A0A140NCI9 | *narK* | Nitrate/nitrite transporte |
| A0A140NCF0 | *narZ* | nitrate reductase (quinone) |
| A0A140NC85 | *mdtP* | RND efflux system, outer membrane lipoprotein, NodT family precursor |
| A0A140NC74 | *uspE* | UspA domain protein UspA |
| A0A140NC62 | *phnI* | Phosphonate metabolism |
| A0A140NC50 | ECBD_2176 | Flavin reductase domain protein FMN-binding |
| A0A140NBX8 | *artJ* | Cationic amino acid ABC transporter, periplasmic binding protein precursor |
| A0A140NBF3 | *paoB* | Molybdopterin dehydrogenase FAD-binding |
| A0A140NBC0 | *radA* | DNA repair protein RadA |
| A0A140NBB8 | *ssuB* | ABC transporter related |
| A0A140NAZ3 | *dacD* | serine-type D-Ala-D-Ala carboxypeptidase precursor |
| A0A140NAR9 | *ruvB* | Holliday junction ATP-dependent DNA helicase RuvB Holliday |
| A0A140NAN4 | *wzzB* | Lipopolysaccharide biosynthesis protein白 |
| A0A140NAE6 | ECBD_2307 | Extracellular solute-binding protein family 1 precursor |
| A0A140NA22 | ECBD_3157 | RHS protein |
| A0A140N9Y2 | *ydeP* | Oxidoreductase alpha (Molybdopterin) subunit |
| A0A140N9U8 | *acrD* | Efflux pump membrane transporter precursor |
| A0A140N9H2 | *dsdA* | D-serine dehydratase |
| A0A140N990 | *ascB* | Glycoside hydrolase family 1 |
| A0A140N956 | *mdoH* | Glucans biosynthesis glucosyltransferase H |
| A0A140N911 | ECBD_1163*, ECBD_1386* | Integrase catalytic region |
| A0A140N8W2 | *bluF* | Diguanylate phosphodiesterase |
| A0A140N8P7 | *thiM* | Hydroxyethylthiazole kinase |
| A0A140N8H2 | *yejK* | Nucleoid-associated protein YejK |
| A0A140N8D7 | *thiD* | Phosphomethylpyrimidine kinase |
| A0A140N898 | ECBD_0892 | Integral membrane protein TerC |
| A0A140N893 | *ldhA* | D-isomer specific 2-hydroxyacid dehydrogenase NAD-binding |
| A0A140N890 | *ygeA* | L-aspartate/glutamate-specific racemase L |
| A0A140N802 | *glpQ* | Glycerophosphoryl diester phosphodiesterase precursor |
| A0A140N7T5 | *inaA* | Lipopolysaccharide kinase |
| A0A140N7T1 | *dapE* | Succinyl-diaminopimelate desuccinylase |
| A0A140N7Q0 | ECBD_1309 | Formate/nitrite transporter |
| A0A140N7D4 | *mtr* | Aromatic amino acid permease |
| A0A140N7B5 | *narW* | Nitrate reductase molybdenum cofactor assembly chaperone |
| A0A140N788 | *gltD* | Glutamate synthase, small subunit |
| A0A140N771 | *sspB* | Stringent starvation protein B |
| A0A140N723 | ECBD_0294 | Glutathione hydrolase proenzyme precursor |
| A0A140N6U5 | *pdeH* | Diguanylate phosphodiesterase |
| A0A140N6I6 | *nikA* | Nickel ABC transporter, periplasmic nickel-binding protein precursor |
| A0A140N6F9 | ECBD_1573 | DUF1508 domain-containing protein |
| A0A140N5U1 | *sdaC* | Serine transporter |
| A0A140N5T4 | *gguA* | ABC transporter related |
| A0A140N5S0 | *tdcD* | Propionate kinase |
| A0A140N5K6 | ECBD_0627 | Endoribonuclease L-PSP |
| A0A140N518 | *gspE* | Type II secretion system protein E |
| A0A140N4Z3 | *ypdA* | histidine kinase |
| A0A140N4R8 | *hyfG* | NADH dehydrogenase (Ubiquinone) 30 kDa subunit |
| A0A140N4N5 | *recB* | RecBCD enzyme subunit RecB |
| A0A140N4K7 | *xylF* | D-xylose ABC transporter, periplasmic substrate-binding protein precursor |
| A0A140N4F8 | ECBD_0387 | Filamentation induced by cAMP protein Fic |
| A0A140N4A7 | ECBD_0336 | Ferrous iron transport protein B |
| A0A140N4A3 | ECBD_0054 | Outer membrane autotransporter barrel domain protein precursor |
| A0A140N477 | ECBD_0024 | Sulfatase |
| A0A140N431 | ECBD_0252 | DUF4049 domain-containing protein |
| A0A140N3W0 | *agaB* | PTS system, mannose/fructose/sorbose family, IIB subunit |
| A0A140N3P4 | *waaH* | Glycosyl transferase family 2 |
| A0A140N3N8 | *nanA* | N-acetylneuraminate lyase |
| A0A140N370 | ECBD_0307 | Gluconate transporter precursor |
| A0A140N2E4 | ECBD_0281 | Extracellular ligand-binding receptor precursor |
| A0A140N2D6 | *tusA* | Sulfur carrier protein TusA |
