# Supplementary material for: The effects of NDM-5 on Escherichia coli and the screening of interacting proteins
Source: Front Microbiol. 2024 Jan 30;15:1328572. doi: 10.3389/fmicb.2024.1328572 (PMC10861311; doi:10.3389/fmicb.2024.1328572)
Supplement: Supplementary file 2 [file Data_Sheet_2.doc]

Raw picture:

<https://www.jianguoyun.com/p/DbW-QHwQh8GRDBiflaUFIAA>

Results of transcriptome analysis:

<https://www.jianguoyun.com/p/DeBSq18Qh8GRDBihlaUFIAA>

Raw data:

https://www.jianguoyun.com/p/Df8zlmAQh8GRDBiilaUFIAA
